# Convergence of Multiple MAP3Ks on MKK3 Identifies a Set of Novel Stress MAPK Modules

**DOI:** 10.3389/fpls.2016.01941

**Published:** 2016-12-22

**Authors:** Jean Colcombet, Cécile Sözen, Heribert Hirt

**Affiliations:** ^1^Institute of Plant Sciences Paris-Saclay, Centre National de la Recherche Scientifique, Institut National de la Recherche Agronomique, Université Paris-Sud, Université d’Evry, Université Paris-Diderot, Sorbonne Paris-Cité, Université Paris-SaclayOrsay, France; ^2^Center for Desert Agriculture, King Abdullah University of Science and TechnologyThuwal, Saudi Arabia

**Keywords:** viridiplantae, MKK3 module, phosphorylation cascade, stress responses, mitogen-activated protein kinases

## Abstract

Since its first description in 1995 and functional characterization 12 years later, plant MKK3-type MAP2Ks have emerged as important integrators in plant signaling. Although they have received less attention than the canonical stress-activated mitogen-activated protein kinases (MAPKs), several recent publications shed light on their important roles in plant adaptation to environmental conditions. Nevertheless, the MKK3-related literature is complicated. This review summarizes the current knowledge and discrepancies on MKK3 MAPK modules in plants and highlights the singular role of MKK3 in green plants. In the light of the latest data, we hypothesize a general model that all clade-III MAP3Ks converge on MKK3 and C-group MAPKs, thereby defining a set of novel MAPK modules which are activated by stresses and internal signals through the transcriptional regulation of *MAP3K* genes.

## Introduction

Mitogen-activated protein kinase (MAPK) modules are important signaling actors found in all eukaryotic cells. They are constituted of the MAPK *per se*, a serine/threonine kinase which is phosphorylated and activated by a MAPK Kinase (MAP2K), itself activated by phosphorylation by a MAP2K Kinase (MAP3K). These modules are encoded in plants by large multigenic kinase families. For example, the *Arabidopsis* genome codes for 20 MAPKs, 10 MAP2Ks, and about 80 MAP3Ks (Ichimura et al., 2002). The MAPK and MAP2K families are both organized in four well-defined sub-clades (denoted as clades A–D). Under the generic name, MAP3Ks gather several distinct kinase families which were shown in animals to activate MAP2Ks. These include the MEKK-like kinases (20 members in *Arabidopsis*) which are unambiguously involved in the activation of MAP2Ks/MAPKs in plants, the large 48 membered Raf family for which functional data are scarce and the 11 ZIK kinases which have not been reported to act upstream MAP2Ks in plants so far.

Three modules defined by the three iconic MAPKs, MPK3, MPK4, and MPK6, were the focus of the vast majority of MAPK studies (Colcombet and Hirt, 2008; Suarez-Rodriguez et al., 2010). Although some reports exist on the majority of MAPKs and MAP2Ks, plant MKK3s have been the subject of less attention.

## MKK3, An Atypical MAP2K, Found in All Plant Species

The first *MKK3*-like *MAP2K* sequence, *NtNPK2*, was identified from a tobacco cell suspension cDNA library twenty years ago and proved to code for an active kinase (Shibata et al., 1995). Classical MAP2Ks are usually rather small (about 350 amino acids) and are globular proteins with only a single kinase domain. In contrast, NtNPK2 and all MKK3 homologs are considerably longer (>500 amino acids) and contain, besides the protein kinase domain, a NUCLEAR TRANSPORT FACTOR2 (NTF2)-like domain in their C-terminal regions. The MAP2K N-terminal tail, which contains a docking site (D-site) for MAPKs, is also well conserved within MKK3 homologues but divergent from those of other MAP2Ks. Additionally, MKK3 N-terminal tails have secondary structure motifs which are usually not found in other MAP2Ks.

In the *Arabidopsis* genome, there is a single *MKK3* gene (At5g40440) which defines the B subclade of plant MAP2Ks (Ichimura et al., 2002). In the published plant genomes, B-type MAP2Ks are found in almost all dicots and monocots as well as in the primitive angiosperm *Amborella trichopoda* as a single copy gene. The two gymnosperm sequenced genomes (*Pinus taeda* and *Picea abies*) do not seem to contain any MKK3 but the explanation could also be due to their low sequence coverage. Alternatively, a loss of MKK3 in gymnosperms could be possible. The moss *Physcomitrella patens* possesses two well-defined MKK3-like kinases (PpMKK3-1 and PpMKK3-2). The unicellular green algae *Chlamydomonas reinhardtii* codes for very few genes having homology to *MAP2Ks*. Singularly, the best MAP2K candidate [referred as MAP Kinase Kinase 1 in NCBI (XP_001696437) and CrMKK3 in this article] possesses an NTF2-like domain in its C-terminus. Overall, the *CrMKK3* sequence is not very similar to other MKK3s but the *CrMKK3* gene shares with *AtMKK3* several conserved introns which are not found in other *Arabidopsis* MAP2Ks, suggesting that *CrMKK3* and *AtMKK3* have a common ancestor containing an NTF2 domain and that the primary gene structure is highly conserved during evolution. This also suggests that the MKK3-related pathways are conserved among all photosynthetic eukaryotes.

At present, a role of the NTF2 domain in plant MKK3s has not been reported. A NUCLEAR TRANSPORT FACTOR2 domain suggests a function in the localization or shuttling of the protein kinase. In eukaryotes, NTF2s are small proteins which have indeed been involved in protein import into the nucleus (Moore and Blobel, 1994) and play an important role in the β-importin-dependent macromolecular trafficking by loading the nucleus with RanGTP (Stewart et al., 1998). But other proteins containing an NTF2-like domain have distinct roles. For example, metazoan p15, through interaction with NTF2s is an important actor of nuclear mRNA export (Fribourg et al., 2001; Stutz and Izaurralde, 2003). These data indeed suggest that MKK3 signaling could require specific shuttling mechanisms between cellular compartments. However, Ca^2+^/calmodulin dependent protein kinase II (CAMKII) also possesses in its C-terminal tail an NTF2-like domain which has been shown to be involved in its assembly of dodecamers (Morris and Török, 2001; Rellos et al., 2010). Therefore, the NTF2 domain might also function in homo- or oligomerization of MKK3 proteins. But some NTF2-like domains may also have catalytic activities. For example, the NTF2-like *Streptomyces* SnoaL codes for a small polyketide cyclase involved in antibiotic synthesis and the bacterial δ5-3-ketosteroid isomerase catalyzes the stereospecific isomerization of steroids (Kim et al., 1997; Sultana et al., 2004), opening up the possibility that the NTF2-like domain of plant MKK3s might also have an enzymatic activity which would be coupled to MAPK signaling. Structure-function investigations would be necessary to elucidate the exact role of this mysterious domain.

## In Plants, MKK3 has Been Involved in Several MAPK Modules and Responses to Environment

**FIGURE 1:**
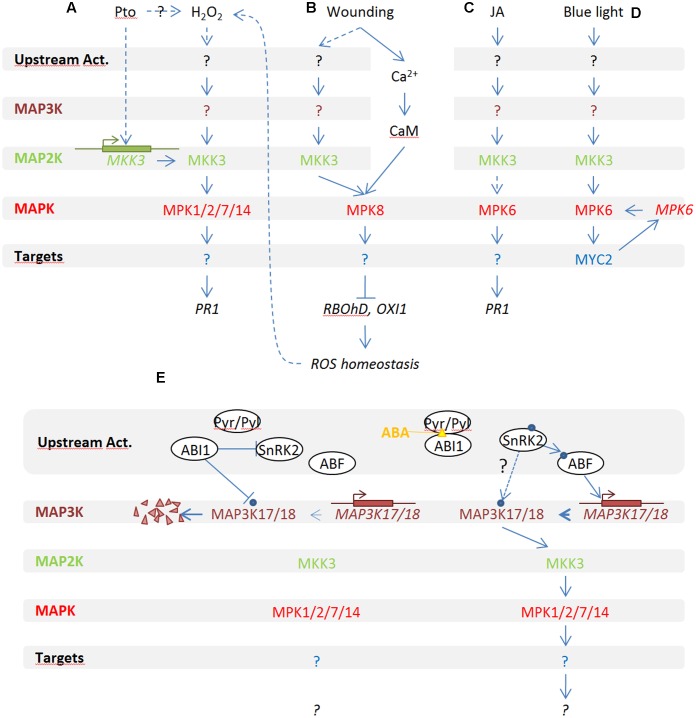
**Overview of MKK3 implication in MAPK modules in *Arabidopsis thaliana***. MKK3 functions upstream of MPK1/2/7/17 in response to H_2_O_2_ **(A)** and ABA **(E)**, of MPK8 in response to wounding **(B)** and with MPK6 in response to jasmonic acid (JA) **(C)** and red light **(D)**.

In plants and mainly in *Arabidopsis*, several functional studies placed MKK3 within MAPK modules highlighting its function in several distinct physiological signaling processes. **Figure 1** gives an overview of our actual knowledge about MKK3 modules in physiological processes.

### MKK3 in ROS Signaling and Homeostasis

MKK3 was first reported to play a role during plant–pathogen interaction (Dóczi et al., 2007) (**Figure 1A**): *mkk3* knock-out mutants were shown to be hypersensitive to infection by *Pseudomonas syringae* DC3000 whereas *Arabidopsis* lines expressing a constitutively active MKK3 (MKK3EE) showed mild resistance to the pathogen. Using yeast two hybrid and co-immunoprecipitation assays from protoplasts, this study also suggested that MKK3 and the four C-group MAPKs MPK1/2/7/14 interact together to define a functional MAPK module, that can be activated by H_2_O_2_. In support of this notion, MPK1/2 activation by H_2_O_2_ has been confirmed *in vivo* by an independent study (Ortiz-Masia et al., 2007).

Interestingly, an MKK3-based MAPK module has also been reported to act upstream of reactive oxygen species (ROS) production in response to wounding (Takahashi et al., 2011) (**Figure 1B**). In this study, the authors showed that plants mutated in *MPK8*, which codes for a MAPK of the poorly characterized D-clade, fail to locally restrict ROS production in response to leaf wounding. Coherently, MPK8 over-expressing plants do not produce ROS anymore in response to wounding as revealed by DAB staining. The authors also showed that MPK8 activation upon wounding depends on calcium/calmodulin as well as on MKK3. This indirectly suggests that MKK3, which is activated by H_2_O_2_, is also involved in ROS production upon stress conditions such as wounding. To test whether MKK3-MPK8 forms a functional module, it could be relevant to test whether *mkk3* plants are impaired in wound-induced ROS production.

### MAP3K17/18-MKK3-MPK1/2/7/14 in Response to ABA, Senescence, and Dormancy

In *Arabidopsis*, MPK1 and MPK2 were shown to be activated by abscisic acid (ABA) (Ortiz-Masia et al., 2007; Umezawa et al., 2013; Danquah et al., 2015). Recently, two independent studies demonstrated that MKK3, together with MAP3K17 and MAP3K18, functions upstream of MPK1, MPK2, MPK7, and MPK14 in an ABA-activated MAPK module (Danquah et al., 2015; Matsuoka et al., 2015) (**Figure 1E**). Apparent discrepancies between these studies also suggest a dual mode of activation of the ABA-related MKK3 module. Indeed, Danquah et al. (2015) showed that, under their experimental conditions, activation of C-group MAPKs depends on MAP3K18 synthesis, explaining their activation with a rather slow kinetics. Other studies have shown a more rapid activation of the C-group MAPKs which is compatible with a post-translational activation of MAP3Ks (Ortiz-Masia et al., 2007; Umezawa et al., 2013). Interestingly, using lines constitutively expressing tagged MAP3K18, Matsuoka et al. (2015) showed that ABA is also able to directly activate the kinase within 15 min and that MAP3K18 activity peaks at 30 min. Taken together, this suggests that the pathway has two modes of functioning, which may be dependent on unidentified experimental differences between the laboratories working on this ABA-dependent MAPK pathway. Finally, a direct interaction between MAP3K18 and the PP2C phosphatase ABI1, but not with ABI2, has been described (Mitula et al., 2015). The authors hypothesized that besides being one of the main actors of the ABA core signaling module, ABI1 is also able to dephosphorylate MAP3K18 in the absence of ABA, resulting in the degradation of MAP3K18 by the proteasome. When ABA is perceived by PYR/PYL receptors, ABI1 is dissociated from MAP3K18 and MAP3K18 is stabilized and able to activate downstream factors of the signaling module.

Phenotypical characterization in plants with impaired MKK3 module function confirmed the role of MKK3 in ABA signaling and/or ABA-dependent processes. Danquah et al. (2015) characterized mutant plants in a drought and abiotic stress context and showed that plants impaired in the MKK3 module are less able to restrict water loss when submitted to a long-term mild drought experiment. More directly, the stomata of *map3K18* plants are compromised to close in response to ABA (Mitula et al., 2015) whereas *MAP3K18* over-expressors show accelerated leaf senescence (Matsuoka et al., 2015), a process known to be under control of ABA (Song et al., 2016). A very recent study similarly reported that *Nicotiana benthamiana* plants over-expressing the cotton (*Gossypium hirsutum*) *GhMKK3* were more resistant to drought and closed more efficiently their stomata in response to ABA (Wang et al., 2016). Additionally, by QTL mapping for seed dormancy, two recessive mutations in *MKK3* orthologs have been identified in both wheat and barley (Nakamura et al., 2016; Torada et al., 2016). In plants, dormancy is under control of seed hormonal content during maturation and desiccation, with one of the main hormones being ABA (Shu et al., 2016). In the case of the barley mutant cultivar “Azumamugi,” the recessive mutation decreases MKK3 activity and thereby increases seed dormancy, suggesting that the activation of the MKK3 module releases seed dormancy (Nakamura et al., 2016).

### MKK3-MPK6-MYC2 Is Involved in JA and BL Signaling

MKK3 has also been reported to work upstream of MPK6 in the context of two pathways (**Figures 1C,D**). A first study showed that the MKK3-MPK6 module plays a central role in jasmonic acid (JA)-induced root growth inhibition (Takahashi et al., 2007). MPK6 is activated by JA within 10 min and this activation is impaired in *mkk3* but not in *mkk2* mutant plants. Since MPK6 is very efficiently activated by many stresses, including touch, it is unfortunate that mock samples were not tested in these JA-dependent MPK6 activation assays. Coherently, the authors showed that both *mkk3* and *mpk6* mutants are slightly more sensitive to exogenous JA at the root elongation level as well as show reduced activation of the JA-marker gene *PDF1*.

MKK3-MPK6 has also been implicated in blue light (BL) signaling. MPK6 is activated specifically by BL but not red light. Using knock-out mutants, the authors showed that this activation is MKK3- and MYC2-dependent. Lee also reported that an *mkk3* KO mutant shows insensitivity to red light and hypersensitivity to gibberellin (Lee, 2015). Interestingly, it has also recently been shown that MPK6 was able to both interact with the *MYC2* promoter to induce its transcription and to phosphorylate MYC2 (Sethi et al., 2014). Interestingly, this module was shown to also negatively regulate the expression of the *MYC2* transcription factor which is a well-known actor of JA signaling.

### How to Conciliate Apparently Opposite Data

Taking advantage of the yeast 2 hybrid interaction assay, several groups reported on the interactions between MKK3 and the MAPKs encoded by the *Arabidopsis* genome (Dóczi et al., 2007; Lee et al., 2008; Matsuoka et al., 2015). In these studies, MKK3 interacted with C-group MAPKs but not with MPK6 or MPK8. The absence of an MKK3-MPK6 interaction was not due to technical problems as MPK6 was otherwise able to interact with its native MAP2Ks MKK4, and MKK5 (Lee et al., 2008). In protoplast activation assays, a constitutively active MKK3 was also not able to activate MPK6 or MPK8 (Danquah et al., 2015) and no direct interaction between MKK3 and MPK6 could be shown in the studies reporting that MKK3 and MPK6 constitute a functional MAPK module (Takahashi et al., 2007; Sethi et al., 2014). The only suggestion that MKK3 could activate directly MPK6 came from an *in vitro* kinase assay in which a constitutively active MKK3 was able to trigger MPK6-dependent phosphorylation of MBP (Takahashi et al., 2007).

From these results, it is tempting to hypothesize that MKK3 does not interact with MPK6 or MPK8, suggesting that the MKK3-dependent activation of MPK6 may be indirect. The fact that MPK1/2, which have been shown to function downstream of MKK3 in response to ROS and ABA, are also activated by JA (Ortiz-Masia et al., 2007) would promote a model in which the MKK3-MPK1/2/7/14 module is necessary for the proper functioning of the upstream players of JA- or BL-dependent MPK6 activation. The investigation of JA-dependent MPK6 activation in mutants impaired in *MAPK* genes of the C-clade would help to resolve this issue.

## Are Map3Ks of Subclade III Activators of MKK3 Modules?

### MAP3K13-20 Define a Subclade of MEKK-Like Kinases Which Are Strongly Transcribed in Response to Stresses

**FIGURE 2:**
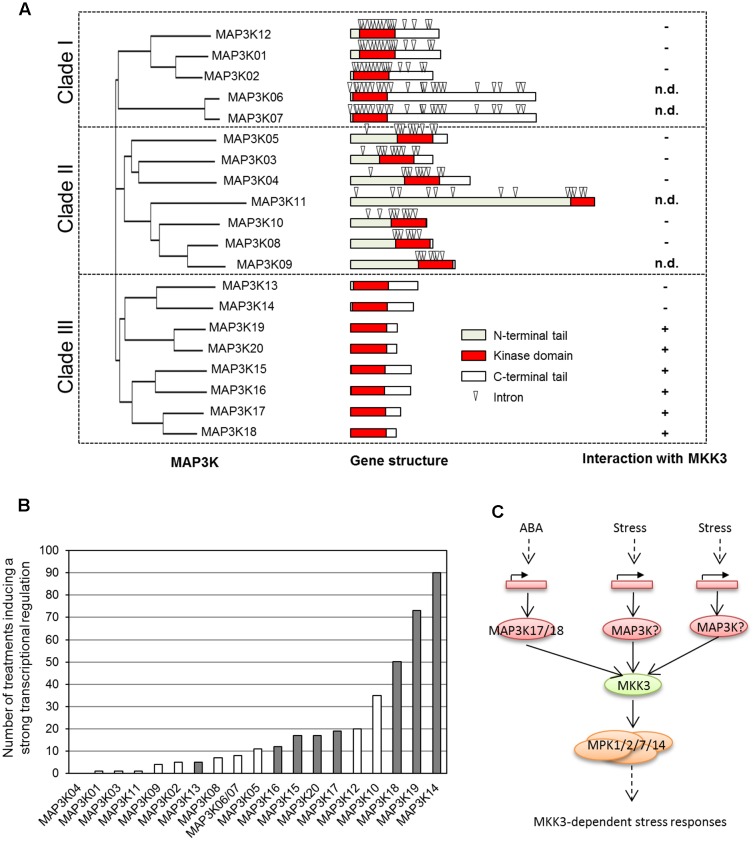
**Are MAP3K of clade III building a functional module with MKK3-MPK1/2/7/14? (A)** MEKK-like MAP3Ks are organized in three subclades with distinct locus organization (adapted from Danquah et al., 2015). Only members of clade III are interacting with MKK3 (determined using yeast 2 hybrid assay). n.d., not determined. **(B)** Number of conditions reported in Genevestigator as able to transcriptionally regulate MAP3K genes (fold-change > 3, P-value < 0.001). MAP3Ks of clade-III are shown in gray. **(C)** General working model for the activation of MKK3-MPK1/2/7/14 module by the stress-dependent transcriptional regulation of MAP3Ks of clade-III.

ZIK-, Raf-, and MEKK-like kinases have been shown to act as MAP2K Kinases in animal cells. In plants, ZIK-like kinases (11 members in *Arabidopsis*) have not been connected to MAPK signaling so far and Raf-like kinases (about 50 members), such as CTR1 and EDR1, seem to act rather as negative regulators of MAPK modules (Yoo et al., 2008; Zhao et al., 2014). In contrast, several MEKK1-like kinases have been shown to be activators of MAP2Ks (Colcombet and Hirt, 2008). The 20 members of this family are organized in three subclades (**Figure 2A**). Genes of subclades I and II have many introns and code for kinases carrying an additional long C- and N-terminus, respectively. Interestingly, MEKK-like kinases of subclade III do not have any introns and code for kinases with rather short tails.

MAP3Ks of subclade III are largely uncharacterized, with the notable exception of MAP3K18 which has been shown to be an important player of ABA signaling (Danquah et al., 2015). This work also showed that in contrary to other MAP3Ks which are thought to be mainly regulated by phosphorylation, *MAP3K18* is strongly transcriptionally regulated in response to ABA and the consequent kinase production triggers the phosphorylation cascade, explaining the delayed activation kinetics of its downstream target MAPKs MPK1, 2, 7, and 14 (Boudsocq et al., 2015; Danquah et al., 2015). Interestingly, we noticed that other members of subclade III are also transcriptionally regulated (Danquah et al., 2015). For example, *MAP3K13* and *MAP3K14* have been shown to be induced upon nitrate feeding after a starvation period (Marchive et al., 2013). Analysis of the Genevestigator database, which integrates many Affymetrix-based transcriptome experiments, confirms that *MAP3K* genes from clade III are under transcriptional regulation by various stresses (**Figure 2B**). This suggests that a transcriptional regulation, as shown in the case of ABA regulation of MAP3K18, might be a general feature of the regulation of clade III *MAP3Ks*.

### Toward a General Model for a Slow Activation of MKK3 Modules by Stresses

In yeast 2 hybrid assays, MKK3 was shown to interact with six of the eight MAP3Ks belonging to clade III but none of the MAP3Ks of clades I and II (**Figure 2A**) (Danquah, 2013; Danquah et al., 2015). Interestingly, clade-III MAP3Ks as well as type-C MAPKs were found in all species containing MKK3. In *Arabidopsis*, MPK1 and MPK2 have been shown to be activated by several stresses, such as wounding, ABA, JA, and H_2_O_2_.. We propose a general model in which clade-III MAP3Ks, MKK3, and C-type MAPKs define functional modules activated by many stresses, one of the main mechanisms of activation being the transcriptional regulation of the upstream MAP3Ks (**Figure 2C**). This obviously does not exclude the possibility that these modules are also directly activated through phosphorylation of these MAP3Ks in response to various stresses.

## Conclusion

Recent evidence suggests that MKK3 modules integrate many signals and represent a new landscape of phosphorylation cascades which deserve further attention as they were recently suggested to be important crop traits in various crops. In the future, besides a functional characterization of these modules, a major challenge will be the identification of the downstream responses controlled by the modules. Similar to the iconic stress-activated MAPKs which are also involved in development, we will have to clarify how MAPKs choose their proper targets when activated by different input signals. A seducing hypothesis is that the MKK3 modules regulate a set of responses which are shared by several signals. Transcriptome experiments using mutants of the different MAP3Ks or the MKK3 hub kinase in response to specific stresses will help to clarify this point and will be of particular interest in the context of the integration of environmental cues.

## Author Contributions

All authors listed, have made substantial, direct and intellectual contribution to the work, and approved it for publication.

## Conflict of Interest Statement

The authors declare that the research was conducted in the absence of any commercial or financial relationships that could be construed as a potential conflict of interest.
